# Recent trends in glycoproteomics by characterization of intact glycopeptides

**DOI:** 10.1007/s00216-023-04592-z

**Published:** 2023-02-22

**Authors:** Susy Piovesana, Chiara Cavaliere, Andrea Cerrato, Aldo Laganà, Carmela Maria Montone, Anna Laura Capriotti

**Affiliations:** Department of Chemistry, Sapienza Università Di Roma, Piazzale Aldo Moro 5, 00185 Rome, Italy

**Keywords:** Glycoproteomics, Intact glycopeptides, Enrichment, Protein glycosylation, Quantitative analysis, Qualitative analysis

## Abstract

This trends article provides an overview of the state of the art in the analysis of intact glycopeptides by proteomics technologies based on LC–MS analysis. A brief description of the main techniques used at the different steps of the analytical workflow is provided, giving special attention to the most recent developments. The topics discussed include the need for dedicated sample preparation for intact glycopeptide purification from complex biological matrices. This section covers the common approaches with a special description of new materials and innovative reversible chemical derivatization strategies, specifically devised for intact glycopeptide analysis or dual enrichment of glycosylation and other post-translational modifications. The approaches are described for the characterization of intact glycopeptide structures by LC–MS and data analysis by bioinformatics for spectra annotation. The last section covers the open challenges in the field of intact glycopeptide analysis. These challenges include the need of a detailed description of the glycopeptide isomerism, the issues with quantitative analysis, and the lack of analytical methods for the large-scale characterization of glycosylation types that remain poorly characterized, such as C-mannosylation and tyrosine O-glycosylation. This bird’s-eye view article provides both a state of the art in the field of intact glycopeptide analysis and open challenges to prompt future research on the topic.

**Graphical Abstract:**

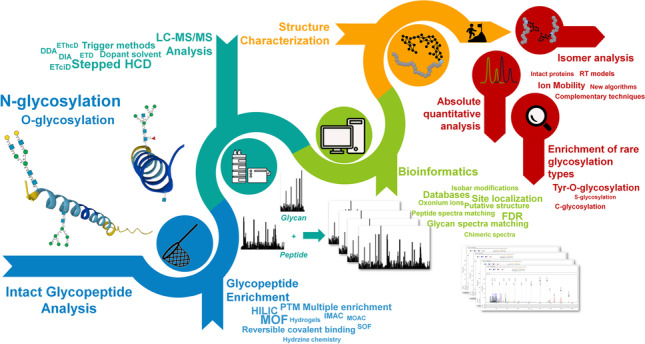

## Introduction

**Fig. 1:**
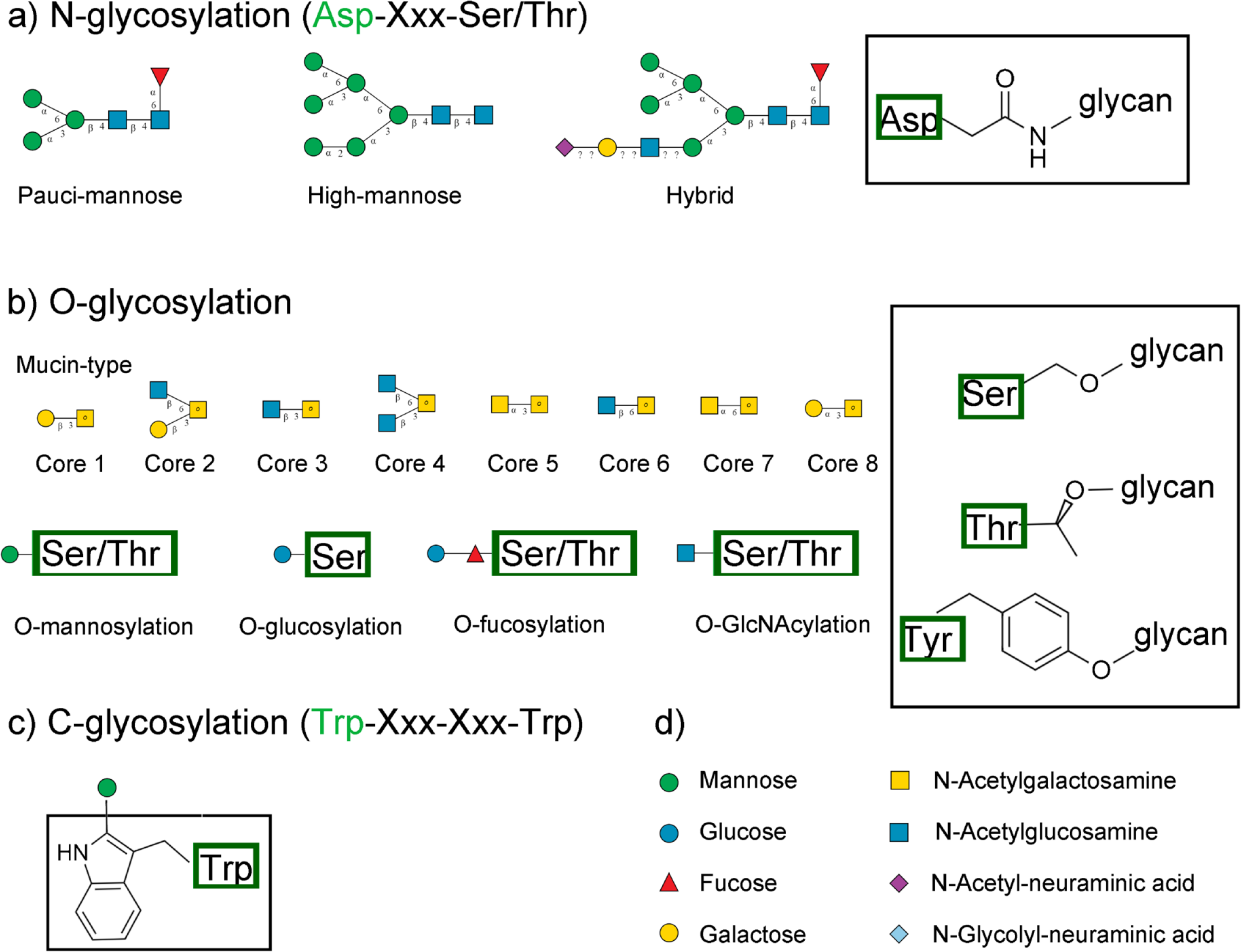
Examples of mammalian N-glycosylations (**a**), O-glycosylations (**b**), C-glycosylation (**c**), with explanation of glycan graphical representation (**d**). Amino acid side chains are shown boxed. Common sequons are also shown, with green residues to mark the glycosite. Structures were drawn using GlycanBuilder2 [3]

Glycosylation is an important protein post-translational modification (PTM), heavily involved in many biological processes, both physiological and pathological ones. For this type of PTM, glycan chains (or single sugars) are covalently linked to the side chains of certain amino acids. Glycan structure is variable, and more types are continuously discovered. Protein N-glycosylation is the most studied one and occurs on asparagine in the sequon Asn-Xxx-Ser/Thr (with Xxx any amino acid different from proline) and, less frequently, in the sequon Asn-Xxx-Cys/Val (Fig. 1a). O-glycosylation occurs on hydroxyl groups mostly of threonine and serine, although it was reported also on tyrosine. Mucin-type O-glycosylation involves the derivatization with N-acetylgalactosamine, which can then be further extended to more elaborate glycan structures, resulting in 8 common core structures that can be further modified and that are the most common type of O-glycosylation in mammalian proteins. O-glycosylation can be very heterogeneous and includes other important classes, such as fucosylation, glycosylation, galactosylation, mannosylation, and N-acetylglucosamination (Fig. 1b). These classes can occur also on completely different residues, such as hydroxylysine. Finally, C-mannosylation is one of the rarest types of glycosylation and occurs on C of tryptophan, in the sequon Trp-Xxx-Xxx-Trp (Fig. 1c) [1]. S-glycosylation has also been reported as N-acetylglucosamine modification of cysteine. It is very rare modification, reported for human and bacteria [2].

**Fig. 2:**
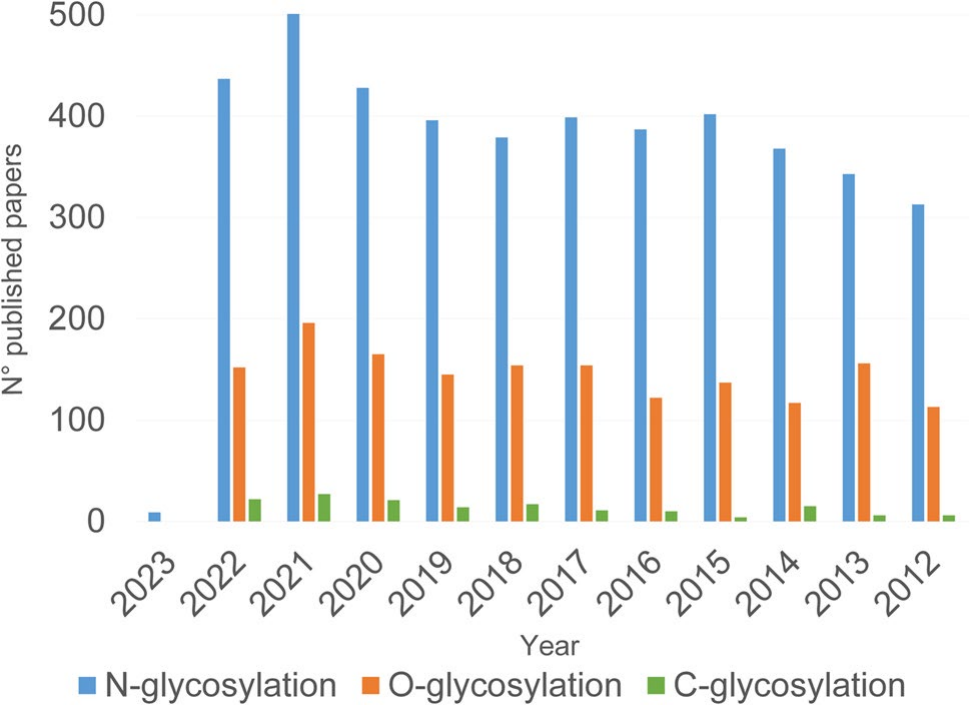
Number of published papers dealing with N-, O-, or C-glycosylation over the last years and resulting in Scopus database accessed in December 2022

Glycoproteomics by liquid-chromatography coupled with mass spectrometry can be performed by studying the released glycans and peptides obtained by deglycosylation or by directly characterizing the intact glycopeptides. Deglycosylation of enriched glycopeptides was the main approach for the indirect and confident identification of glycosites, and it was popular for N-glycosylation analysis. However, by this approach, the connectivity between peptides and the linked glycans is lost, although the LC–MS analysis and spectra annotation process are less complex than in the case of intact glycopeptide analysis. Intact glycopeptides maintain the peptide-glycan link; therefore, they can provide information on the peptide sequence, linked glycan structure, and glycosite [1, 4, 5]. The establishment of mature technologies for the large-scale profiling of thousands of intact glycopeptides required significant efforts and has become affordable due to the technical improvements, which involve the development of dedicated sample enrichment methods, new MS acquisition and fragmentation strategies, and especially new bioinformatics software for confident automated matching of intact glycopeptide spectra [1]. The recent literature witnesses such developments, and there are many reports and review articles on the topic (Fig. 2).

Most of the methods are developed for the analysis of N-glycopeptides, followed by serine and threonine O-glycopeptides, while reports on C-glycosylation, tyrosine O-glycosylation, or S-glycosylation are very scarce.

This trends article aims at summarizing the most recent trends in the analysis of intact glycopeptides, considering the most important steps in the analytical workflow, i.e., the enrichment and selective isolation of intact glycopeptides from complex peptide mixtures, the LC–MS analysis, and the data analysis by bioinformatics software for spectra matching. Reference to the recent literature has been provided and complemented with the latest developments for each discussed topic, with special attention on the open challenges and issues, which need further advances. Considering the literature on intact glycopeptide analysis by shotgun proteomics technologies, this trends article will deal mostly with N- and O-glycosylation types, especially from mammalian samples.

## Enrichment methods for intact glycopeptide analysis

Digestion does not usually require special needs for analysis of intact glycopeptides, and trypsin is the most common enzyme used for digestion in intact glycopeptide analysis as in ordinary bottom-up shotgun proteomics, except for densely O-linked glycosylated regions that are resistant to tryptic digestion due to lack of cleavage sites. In this case, dedicated protocols using other proteases are used, including chymotrypsin, endoproteinase GluC, and endoproteinase AspN [6, 7]. The selective enrichment of glycopeptides is often needed to obtain the maximum amount of analytical information. Most enrichment methods were initially coupled with deglycosylation, but can nonetheless be extended to intact glycopeptide analysis if the enrichment can be performed without disrupting the glycan-peptide link [8]. A brief overview of the most important enrichment strategies is provided in the following sections, with special attention to the new trends in the field.

### Lectins

The use of lectins is compatible with intact glycopeptide analysis. Lectins have been used in affinity chromatography due to the capability of recognizing glycosylation patterns in glycoproteins. Several types of lectins and supports are commercially available and allow enrichment by solid-phase extraction, magnetic solid-phase extraction, and dispersive solid-phase extraction. Non-commercial materials functionalized with lectins are also popular with additional applications, including online enrichment cartridges, and filter-assisted enrichment, where the separation is achieved by using lectin-functionalized sorbents or free lectins on molecular weight cut-off membranes. Compared to other enriching materials, lectins can be very specific and can enrich single sugars, which would be otherwise difficult to recognize by chemical methods, for instance, fucose. Such specificity can hinder the study of comprehensive glycosylation profiles, although the coverage can be improved by the combination of different types of lectins. Some popular lectins include, and are not limited to, concanavalin A, wheat germ agglutinin, *Sambucus nigra*, *Ricinus communis* agglutinin, and jacalin. Lectin affinity has been extensively used for enrichment of N-glycosylation and O-glycosylation. It is an established approach; therefore, we refer the reader to dedicated review articles for further details and applications [8, 9].

### HILIC materials

Hydrophilic interaction liquid chromatography (HILIC) materials are emerging as selective enrichment phases for intact glycopeptide sample preparation. The interaction occurs between linked sugars and the water-rich layer on the polar material, without sequence selection; therefore, it is applicable in large-scale glycoproteomics analysis [8], including quantitative analysis [10]. HILIC materials can exploit different types of preparation procedures and belong to different types of materials, such as bare silica, functionalized silica, or other types of particles (including magnetic materials), and polymers, among the most common ones. A polar functional group is needed for HILIC, and it can be neutral or charged, such as sugars, amino acids, amides, amines, metal–organic frameworks (MOFs), and covalent organic frameworks (COFs). All these materials and their applications for glycopeptide analysis have been recently summarized elsewhere [11]. In HILIC applications, the use of hydrogels for preparation of new materials has been recently suggested for N-glycopeptide sample preparation. Magnetic graphene was functionalized with poly(carboxybetaine acrylamide) to produce a zwitterionic polymer able to combine with water through electrostatic-induced hydration to form a super-hydrophilic hydrogel, which was very sensitive for recovery of glycopeptides from both standard protein digests and serum [12].

### Organic frameworks

MOFs are nanomaterials where metal ions and organic self-assemble into organic–inorganic hybrids by coordination bonding or inter-molecular forces to form three-dimensional crystalline structures. Due to their structural diversity, MOFs are special nanomaterials characterized by a large specific surface area and porosity. The most popular MOFs used in sample preparation, including glycopeptide enrichment, are based on zeolitic imidazolate frameworks (ZIF), materials of institute Lavoisier frameworks (MIL), and the University of Oslo (UiO) series. MOFs have been used for the enrichment of glycopeptides, mostly by exploiting their derivatization with polar moieties to produce HILIC materials. MOF materials and MOF composites are often exploited in magnetic solid-phase extraction protocols. Recent applications of MOFs exploit additional post-synthesis modifications to increase the hydrophilic properties by the covalent link of sugars, boric acid derivatives, and amino acids [13–16]. For instance, the polyethyleneimine-ZIF-8 MOF was functionalized with L-glutathione and gold nanoparticles, to provide hydrophilic sulfhydryl groups and affinity by Au. The performance indicated high sensitivity (down to 2 fmol) and selectivity and was finally tested on serum [17]. UiO-66-NH_2_ MOF preparation was modified using phytic acid as a partial substitution of 2-aminoterephthalic acid ligands to significantly improve the hydrophilicity and affinity for glycopeptides [18].

As stated in the previous section, recently hydrogels have been introduced as materials suitable for HILIC. A MOF hydrogel composite made up of Zn^2+^, polyethyleneimine, and 2-methylimidazole (denoted as ZIF-8/SAP) was recently described. The material, which was characterized by a stable three-dimensional (3D) network structure and an excellent hydrophilicity, allowed the enrichment of N-glycopeptides with high selectivity and sensitivity, suitable for analysis of serum [19].

COFs are periodic and crystalline organic porous polymers with increased applications in bioanalytical chemistry, due to their high surface area, intrinsic porosity, abundant binding sites, and better chemical and thermal stability than MOFs due to the strong covalent bonds between light elements. They can also be post-synthesis modified, to add functional groups for multiple interactions. Despite the advantages, their application to glycopeptide enrichment is relatively scarce compared to MOF, but some applications have been described. For instance, the COF of 1,3,5-triformylbenzene and 3,3-dihydroxybenzidine was prepared on magnetic nanoparticles, then derivatized with 3-glycidoxypropyltrimethoxysilane and polyethyleneimine for improved hydrophilicity, and finally with boric acid for selectivity. It provided good selectivity and was finally applied to saliva [20].

### Use of reversible chemical derivatization

**Fig. 3:**
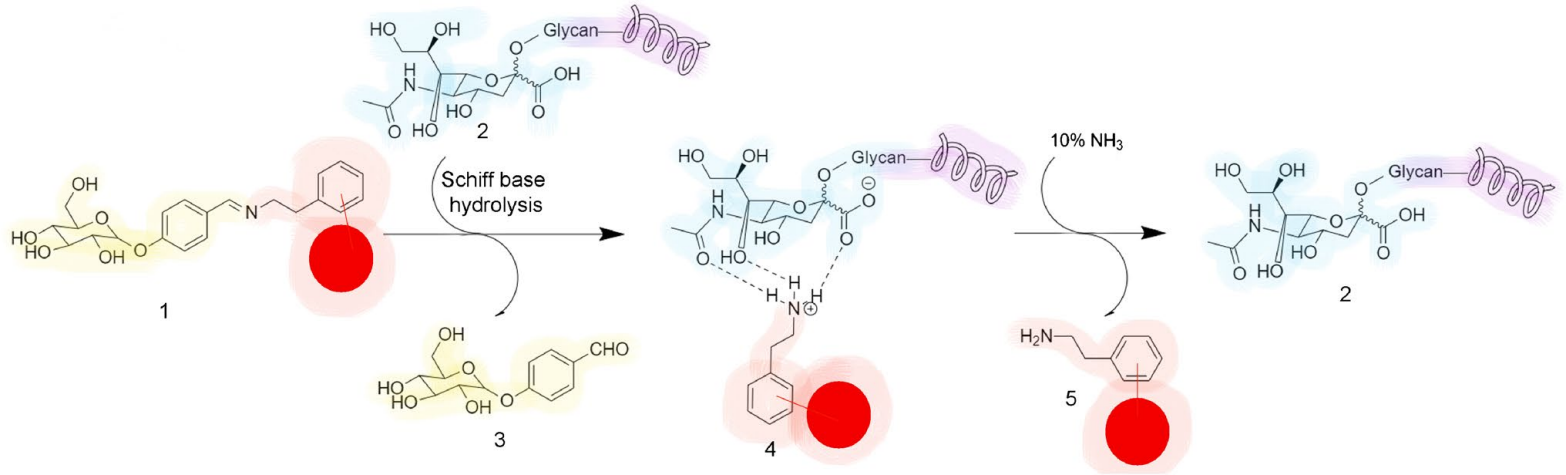
Graphic representation of the enrichment mechanism of sialylated glycopeptides by reversible binding. The support (red) has a linked glucose moiety through Schiff base (yellow). This material (1) can enrich sialylated glycopeptides (2) by hydrolysis of the Schiff base due to reaction with the sialic acid (blue) in the glycopeptide (purple). The hydrolysis of the Schiff base releases the linked glucose (3) and results in the formation of a complex (4) between the protonated amine of the stationary phase and the sialylated glycan. The complex is finally disrupted by ammonia upon elution and releases the support (5) and the intact sialylated glycopeptide (2)

Chemical derivatization can be used for the enrichment and analysis of intact glycopeptides when the covalent derivatization is reversible. One very popular strategy exploits boronic acid selective reaction with cis-diols to form boronate esters, which are easily cleaved under acidic conditions. The bond can be weak and enrichment not efficient for low abundance glycopeptides; therefore, several composite materials with boronate affinity moieties were developed over the last years, including magnetic ones [8, 21]. Other interesting reversible reactions were reported recently, such as Schiff base derivatization. This strategy was described for the enrichment of sialylated peptides, which can hydrolyze the Schiff base and form a complex with the released protonated amine group by ionic and hydrogen bond interactions. The method was selective because the carboxylic moieties on amino acid side chains cannot hydrolyze the Schiff base. The complex can then be disrupted by elution with an ammonia solution to release the intact glycopeptide (Fig. 3) [22].

The selectivity was assessed on tryptic digests, so the presence of acidic side chains did not interfere with the enrichment process. Despite the excellent performance, the enrichment material needed a complicated process for production, and each time a characterization was also necessary.

**Fig. 4:**
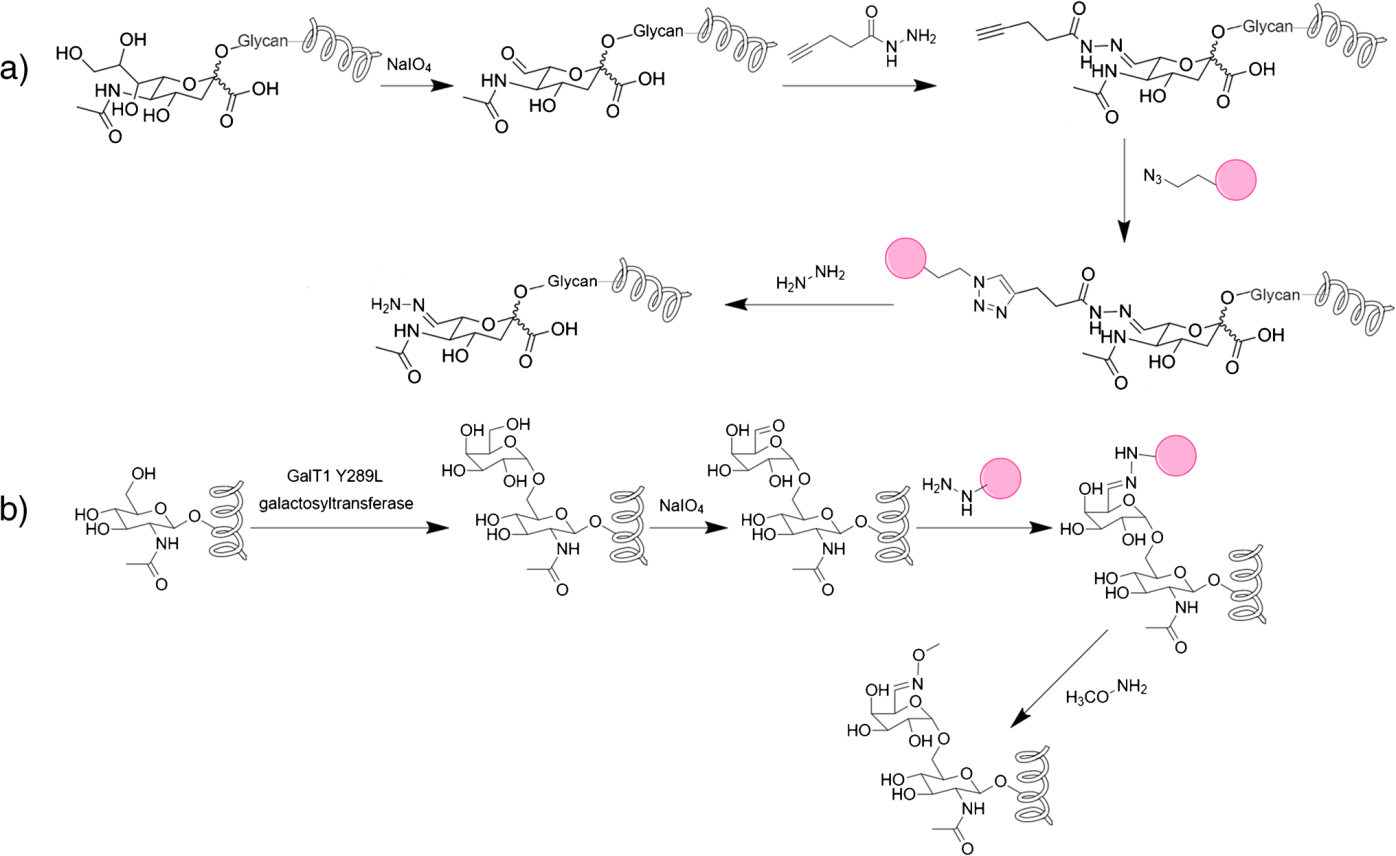
Reversible chemical derivatization for the enrichment of N-linked glycopeptides (**a**) and O-GlcNacetylation glycopeptides (**b**) using hydrazine chemistry

Other traditional methods coupled with deglycosylation analysis can still be adapted to the analysis of intact glycopeptides. Hydrazide chemistry was extensively used in the past and exploited the selective oxidation of cis-diols to react them with hydrazine derivatives linked to a solid support. The peptides were then released by hydrolysis with deglycosylation using PNGase F. The method is particularly suitable for N-glycopeptide analysis, and it was recently modified to allow retention of the peptide-glycan link, although leaving a tag where the derivatization occurred. The hydrazide reagent was immobilized on solid support by click chemistry. At the same time, the hydrazide could react with oxidized sialylated glycans to provide a hydrazone, which could undergo dynamic exchange in excess of hydrazine, thus releasing the linked glycan and leaving a fixed modification as a tag on the intact glycopeptide [23]. This approach is particularly interesting because enrichment is achieved in a homogeneous phase, and the derivatization leaves a fixed tag (Fig. 4a).

A similar strategy was described for the enrichment of O-GlcNacetylation [24]. As the efficiency of oxidation is low for these structures, an improved strategy was developed exploiting enzymatic derivatization with galactose, followed by the typical oxidation with NaIO_4_ (Fig. 4b). This is much more efficient than direct chemical oxidation and provided two aldehyde moieties suitable for derivatization with hydrazide beads. In both these examples the native glycan is covalently modified at the end of the glycan chain in a known way that must be considered in data analysis by modification of suitable glycans in the database or by introduction of a suitable variable modification for database search.

## Dual enrichment of glycopeptides and other modifications

From the analytical method perspective, dual enrichment has the advantage to spare rare samples and LC–MS/MS runs, with a gain in both experimental time and costs, which are features making the method more efficient and with higher throughput. The analysis of more than one PTM has a very important biological significance as well, because it allows shedding light on PTM crosstalk, i.e., the combinatorial action of multiple PTMs on the same or on different proteins for higher order regulation [25].

As far as glycopeptides are concerned, methods for the simultaneous enrichment with other PTMs were described, especially with phosphorylation. Joined enrichment is possible by exploiting similar enrichment mechanisms. Metal oxide affinity chromatography (MOAC) and immobilized metal affinity chromatography (IMAC) materials enrich negatively charged phosphopeptides by exploiting their affinity to metal oxides or metal cations, respectively. These materials can also enrich glycopeptides with negatively charged glycans, i.e., sialylated glycopeptides [8].

Dual enrichment can also be achieved by producing elaborate composite materials with multiple functions, such as MOF materials combining IMAC or MOAC interactions with HILIC. MOFs naturally contain metals suitable for the enrichment of phosphopeptides, including zirconium. An alternative strategy for dual enrichment exploits the combination of IMAC or MOAC with boronic acid chemistry. Compared with SiO_2_- and TiO_2_-based functional nanomaterials that require multiple modifications, “one-step synthesis for two purposes” is one distinct advantage of MOFs [16]. Examples of MOF materials using these strategies were recently reviewed elsewhere [13, 14, 16]. Other recent examples of this approach have been provided by a flexible and hierarchical MOF composite suitable for affinity-tip enrichment. The MOF UiO-66-NH_2_ was grown on the surface of polydopamine-coated elastic melamine foam sponge. The sponge provided a good mechanical stability for tip packing; the MOF was used for enrichment for both phosphopeptides by IMAC and glycopeptides by HILIC, using two dedicated washing/elution protocols. The material was finally applied to saliva [26]. In another example, a composite material was prepared using ZIF-8 grown on a graphene oxide-chitosan foam. The enrichment exploited HILIC and IMAC interaction and was simultaneous for glycopeptides and phosphopeptides, with separate elutions. Good results were obtained from standard protein and serum digests [27].

Supramolecular-organic frameworks (SOFs) are organic frameworks consisting of supramolecular-containing units through noncovalent interactions. Compared to MOFs, they provide additional features, including a simple synthesis, reversible dissolution and processing, easy modification, and controllable assembly. A magnetic SOF combining HILIC with IMAC was recently described for dual enrichment of glyco- and phosphopeptides. The bifunctional gallium ion immobilized magnetic pertriflated pillar[5]arene SOF allowed one-step simultaneous enrichment by the strong hydrophilicity, due to the abundant sulfonic acid groups, and IMAC enrichment by gallium ion. The material was used for magnetic solid phase extraction providing high sensitivity (detection limits as low as 0.1 fmol and 0.05 fmol for glycopeptides and phosphopeptides, respectively). The developed method was particularly interesting, as it allowed elution of separated or combined fractions. Good results were obtained from clinical specimens, cell lysates, and mouse liver tissue samples [28].

COFs can be post-synthesis modified, to add functional groups to achieve multiple interactions needed for dual enrichment. Hydrophilic COF formed by 2,4,6-trihydroxybenzene-1,3,5-tricarbaldehyde and 2,5-divinylbenzene-1,4-diamine was prepared on magnetic colloid nanocrystal clusters coated with polyvinyl pyrrolidone and polyethylenimine. Subsequently, 4-(3-(2-(methacryloyloxy)ethyl)-ureido) benzoic acid was used for derivatization via thiol-ene “click” reaction. The composite allowed multiple hydrogen-bonding interactions, for enrichment of phosphopeptides, and hydrophilic interactions, for glycopeptide enrichment. Phosphopeptides and glycopeptides were eluted together. The developed method was demonstrated suitable for the investigation of complex samples, such as rat liver, and clinical applications, to exosomes extracted from plasma of liver cancer patients [29]. In mouse liver, the method had 82% selectivity for peptides with PTMs, and allowed identification of peptides bearing both modifications.

## LC–MS analysis of intact glycopeptides

The same LC–MS coupling setup used in typical shotgun proteomics can also be employed for intact glycopeptide analysis. Reversed-phase chromatography is suitable for both N- and O-glycopeptides, although more polar stationary phases are also common, especially porous graphitic carbon and HILIC, due to the improved selectivity in the separation of N-glycopeptide isomers [6, 7]. Nonetheless, recent works showed that some improvement in the detection of intact glycopeptides could be obtained by changing the source conditions. Dopant solvents in sweep gases can improve the ionization of these analytes and even allow to avoid enrichment. Acetonitrile and acetone were found the most suitable dopant solvents for this purpose, both in standard and tryptic glycopeptides. The approach allowed to boost the sensitivity, but the application is limited to a specific model of nano-electrospray system; therefore, further studies and improvements are needed to allow widespread application of this approach [8].

MS data-dependent acquisition (DDA) methods used in proteomics needed to be adapted to the analysis of intact glycopeptides. Stepped energy MS/MS methods using common fragmentation strategies, such collision-induced dissociation (CID) and higher-energy collisional dissociation (HCD), provide sequential cleavages of both the glycan and peptide bonds, and result in merged spectra [30]. The confirmation of glycan presence on the peptide can be obtained by the abundant production of diagnostic oxonium ions. The fragmentation of glycopeptides can be improved by electron-capture dissociation and electron-transfer dissociation (ETD), which allow the production of c- and z-type product ions from the peptide backbone with intact attached glycan chains. Trigger methods were developed where glycopeptides are selectively fragmented by ETD only when the signature oxonium ions are detected in HCD scans, and it is affordable on modern instrumentation with fast acquisition rates [4]. Finally, better coverage of both the peptide and glycan sequence can be obtained by hybrid fragmentation methods where ETD is combined with HCD or CID (EThcD or ETciD, respectively). In this way, improved sequence coverage and site localization of glycopeptides are achieved when compared with HCD or ETD fragmentations alone [6, 7]. The most popular approaches for intact glycopeptide MS analysis use stepped HCD or EThcD and allowed characterization of thousands of intact glycopeptides in large-scale studies [1]. The issue was also considered recently, in a systematic study addressing both N-glycopeptides and O-glycopeptides. ETD, EThcD, HCD, stepped HCD, and related trigger methods were compared. Results indicated that product dependent HCD and stepped HCD perform nearly equivalent for routine characterization of N-glycopeptides, although the latter generally creates higher quality spectra, and both of them significantly outperform EThcD methods in terms of identifications despite the lower quality of the obtained spectra. EThcD was found fundamental for large-scale analysis of O-glycopeptides including site localization [31].

Although DDA is a very common acquisition method, data-independent acquisition (DIA) can tackle the limited coverage due to characterization of only the most abundant precursors and their semistochastic selection. DIA acquires MS/MS spectra in a wide *m*/*z* range and results in complex chimeric spectra, which require an elaborate data processing, but it can be advantageous to increase the number of identified glycopeptides, especially low-abundance ones, and improve the reproducibility and quantification, with complete possibility of retrospective analysis. As DIA requires fast scan speeds, it is practically compatible only with HCD, with limited possibility of site localization. Following the trend already observed for the study of other PTMs, DIA has been applied to the analysis of intact glycopeptides [32] and usually requires the creation of custom spectral libraries using traditional DDA. DIA represents a valuable strategy especially suited for quantitative method development [33], with possibility of high-throughput by modification of the spectral library with glycan product ions and automatic removal of low quality spectra. The method allowed quantification of 620 intact N-glycopeptides [34]. DIA methods for intact O-glycopeptide analysis were only recently described and also need good quality fragmentation spectra obtained by enrichment and DDA to build custom libraries. Lectins are used for enrichment; therefore, the lack of specificity is complemented with in-silico spectra libraries generated from experimental evidence of preferential fragmentation upon HCD. The method, called Glyco-DIA, was able to allow the quantitative analysis of 268 O-linked glycopeptides from serum without enrichment [35].

Methods using DIA without prior knowledge on the glycosylation of the samples were also described and exploited signature product ions or neutral losses as screening during the full MS scan. One major limitation of this approach is that it cannot provide information on site localization and is limited to only a few oxonium ions [32].

## Bioinformatics for intact glycopeptide spectra annotation

Nowadays, there are many proteomics software solutions for bioinformatics analysis of MS/MS data and automated peptide matching to protein sequence databases. The same level of development is, however, not met for the matching of intact glycopeptides [7]. Software developed for this purpose has been summarized elsewhere [4, 7] and mainly addresses N-glycosylation. Glycans have typical fragmentation patterns whose matching can be automated in a similar way to the fragmentation patterns of peptides. However, it is difficult to control the false discovery rate (FDR) of matched data for both the glycan part and peptide parts of the molecule. Most available software calculates FDRs on peptides, with only a few exceptions where the glycan is also considered [7]. Confidence is a major bottleneck; this issue was recently addressed for N-glycopeptides by inclusion of Y-ions and B-ions (oxonium) from the MS/MS spectrum, to help discriminate between glycans, and by matching the peptide part before the glycan part. This “peptide-first” approach leverages the well-developed capabilities of modern proteomics tools to determine the peptide sequence and reduces the glycan identification portion to distinguishing between a few glycans that match the mass difference from the determined peptide sequence, rather than distinguishing between the complete search list of up to hundreds of possible glycan compositions. The use of a FDR for the glycan assignment allows to specifically and sensitively identify complex intact glycopeptides and control the FDR of both the peptide and glycan parts [36]. A recent comparison of software solutions for intact glycopeptide analysis revealed comparable performance of freeware and commercial products, with similar limitations, especially for matching glycans with similar or identical masses (N-acetyl-neuraminic acid, N-glycolyl-neuraminic acid, multi-fucose, methionine oxidation, cysteine carbamidomethylation). They are frequently mis-annotated by the current search engines, demonstrating the need for improvement of matching of these glycopeptides. The results in terms of specificity (accuracy) and sensitivity (coverage) were also variable with the search engines, indicating that orthogonal searches and pool of results could be useful for a comprehensive glycoproteomics analysis. The settings used for search also contributed to the discrepancy associated with results, especially the post-search filtering stage [37].

Another drawback of software for intact glycopeptide matching was that they are difficult to use without a good knowledge of informatics. In addition, while good software is available for N-glycosylation, the same does not apply to O-glycopeptides, where bioinformatics software must address additional challenges for matching of large linked-glycans, such as mucin-type O-glycopeptides, due to the high microheterogeneity of the glycosylation, lack of sequon for database search, and possibility of multiple glycosylation sites on the same glycopeptide [4]. Another open issue is the accurate site-specific localization of the glycan, particularly for O-glycopeptides [37]. Finally, structure elucidation is still a problem, due to the many isomeric compositions of linked glycans. Although Y-ions can be used to determine the glycan sequence, putative structures are usually provided and generally retrieved from glycan databases [4].

## The complexity of linked glycans needs further improvement in method development for full elucidation of the biological significance

### Development of enrichment methods for rare glycosylation types

C-mannosylation is a rare PTM that is poorly studied by shotgun proteomics. The characterization of this PTM was possible only from intact protein analysis; therefore, the discovery rate of this modification is low as it is mainly hypothesis-driven and requires a dedicated investigation. For example, C-mannosylation was reported on complement factor P [1], where crosstalk was observed with N-glycosylation. Bioinformatics analyses revealed that over 500 human proteins possess the Trp-Xxx-Xxx-Trp consensus sequence (with only the first tryptophan is modified) and are targeted to the secretory pathway, thus meeting the known requirements for tryptophan C-mannosylation. To date, only 24 of these proteins have been confirmed to possess the modification by MS, NMR, and/or crystallography. Non-canonical C-mannosylation has also been reported. As such, there is need for a detailed characterization of this PTM, and only very recently new strategies have been described to provide a picture on the C-glycome using enrichment strategies. Monoclonal antibodies were developed and allowed the enrichment of peptides possessing tryptophan C-mannosylation even from complex mouse brain tissue [38]. The study revealed many new modification sites on proteins throughout the secretory pathway with both the conventional and non-canonical consensus sequences. The limitation of the approach was that a specific amino acid was necessary close to the mannosylated tryptophan because the antibodies had a sequence preference for the EW(Man) epitope [38]. To avoid this drawback, and taken into consideration that mannose is frequently found on other glycosylation types as well, a new method for enrichment of C-mannosylated glycopeptides was suggested by exploiting lectin A from *Burkholderia cenocepacia* bacteria. It did not require any epitope for recognition and was proved effective even in complex tryptic digests of whole cells [39]. The lectin, prepared from *Escherichia coli* cell cultures, was packed in a 3.5-m-long column after conjugation to agarose beads and used for enrichment after deglycosylation from N-linked glycans and preliminary enrichment with a 3-m concanavalin A lectin column. Both these studies pave the way for the development of dedicated enrichment strategies for this poorly studied PTM and demonstrate the need for high-throughput strategies for the enrichment which can bypass a priori hypothesis and allow discovery of non-canonical glycosylation sites.

Tyrosine glycosylation is another modification that has received little attention in the literature for systematic characterization by glycoproteomics using LC–MS. Indeed, few reports described tyrosine glycopeptides and result from studies addressing O-glycosylation by lectin enrichment. This lack of methods for enrichment of tyrosine glycosylation could be the main limiting factor for application of LC–MS/MS characterization. In fact, the development and application of antibodies for tyrosine O-GalNAcylation recently indicated that tyrosine glycosylation is widely expressed in most human tissues and is likely a ubiquitous and underappreciated PTM [40].

### Differentiation of glycan isomerization in glycopeptides

Although MS is a powerful technique for the characterization of glycopeptides, it cannot provide the exact connectivity of sugar moieties, but that information can be very valuable, as it was associated with disease. The combined use of chromatography and MS^n^ methods can provide information on some structures, such as the terminal sialic acid isomers, but not on all combinations, such as fucosylated or bisecting glycan isomers. In addition, glycopeptides with isobaric linked glycans or isomeric glycopeptides have close elution times in typical reversed-phase chromatography; therefore, it is common the observation of chimeric spectra. These spectra result from two or more precursors being selected in the same MS/MS event and complicate the process of spectra matching and glycopeptide identification [7]. In this sense, the use of ion mobility (IM) is a promising additional tool for isomer separation [41]. It can be considered an additional separation dimension, where ions are separated based on their collisional cross-sections. Glycan connectivity can significantly change the conformation of isomers, which in turn affects the ion mobility of these species. Differently from the enrichment during sample preparation, this approach can be exploited for any glycosylation type. Examples of successful application of IM include the separation of intact glycopeptides that differed only in the glycosylation sites. Furthermore, IM analysis of glycopeptide fragments was demonstrated to be an effective strategy to distinguish α2,3 versus α2,6 sialic acid linkages on intact glycopeptides [8, 42]. Much more recently, IM was also used for relative quantitation of the α2,3 or α2,6 sialic acid isomers, using a time window method for sialic acid containing peptides identified in a normal run. The method, developed on haptoglobin, was suitable for serum study and biomarker discovery [43]. IM was successfully applied to both N- and O-glycopeptides [8].

IM was also recently suggested as an alternative strategy to peptide enrichment, because it allows separation and detection of classes that cannot be enriched by common materials. Field asymmetric waveform IM was found suitable for separation of short aliphatic glycopeptides even from complex mixtures. In addition, the use of compensation ramping voltage provided access to both N- and O-glycopeptides in the same experiment [8]. This type of IM was also found suitable for typical bottom-up proteomics studies, even of complex samples [42]. The performance of high-field asymmetric waveform IM has a powerful separation capacity, which was recently compared to that of a 2D-LC system in a typical proteomics workflow. Internal compensation voltage stepping experiments can increase protein identifications from a single-shot experiment to > 8000, from over 100,000 peptide identifications in as little as 5 h, demonstrating it to be compatible with high-throughput analysis [44]. Even if many reports and strategies using IM have been described in the literature for the study of intact glycopeptides, the high-throughput application of this technology is still limited, and future improvements are needed to move from the proof-of-principle stage. Assigning signals to individual structures, with detailed description of stereochemistry, is not straightforward in large-scale investigations. Reasons that hinder comprehensive glycoproteomics isomeric analysis are connected with IM technology, which has not sufficient resolution to achieve complete separation of isomers in complex biological samples or complex structural molecules [41]. The use of collisional cross-sections, as additional criteria for structural identification by database search, also has limitations that prevent application in routine identification. The major drawbacks are the lack of intact glycopeptide standards, which are needed to build libraries, and the variability of collisional cross-sections on the specific instrumental conditions. In addition, IM MS produces complex data that need suitable bioinformatics software for data analysis and processing in a high-throughput and rapid way [42].

### Quantitative analysis of intact glycopeptides

As stated in the previous sections, protein glycosylation is fundamental for biomarker discovery. As such, there is a need not only for qualitative analysis but also for quantitative analysis. Quantification at the full scan level can be obtained using strategies already employed for general shotgun proteomics analysis, involving both label-free or labelling strategies. Moreover, the labelling of sugar moieties was also described, including the isotope-tagged cleavable linker specific for O-glycopeptides. Alternatively, quantitative analysis on product ion spectra can be carried out using labelling strategies as described for proteomics analysis. DIA has also been suggested as an effective and improved quantitative strategy for intact glycopeptides, as described before. These strategies provide relative quantitative data with high throughput and are particularly useful in biomarker discovery. However, the absolute quantification of intact glycopeptides is fundamental to elucidate the biological function in biomedical research and is needed for the validation of global proteomics studies. Traditional multiple reaction monitoring and parallel reaction monitoring analysis from target precursors selected in the discovery analysis (whose *m*/*z* and retention time are known) represents the main approach with lower throughput but higher sensitivity. For intact glycopeptides, the absolute quantitative analysis is still an open issue, due to the shortage of pure glycopeptide standards and the heterogeneity of a single glycosylation site. Surrogate standards, with the same peptide backbone and different glycan, were used to improve the quantitative analysis performance, but intact synthetic standards are still necessary for absolute quantification. Intact glycopeptides possess a peptide and a sugar moiety. The latter provides oxonium ions that are diagnostic of glycosylation and can be used for transition monitoring; however, recent studies demonstrate that the inclusion of Y-ions (generated by partial digestion of the glycan moiety or by soft fragmentation techniques) allows for improvement of both the specificity and the S/N ratio over the use of oxonium ions alone [10]. The use of pure standards is important to confirm the glycan structure and validation. In fact, it was demonstrated that although fragmentation patterns may be very similar between isomeric glycans, the detailed study of product ions’ relative abundance can help shed light on the glycosidic bond isomerism and determine which is the correct structure of a biomarker initially detected by shotgun proteomics [45]. Such resolution of isomeric glycoconjugates is not always possible by MS/MS analysis alone, especially when the number of coexisting isomeric glycans is large. Despite the limited number of studies on intact glycopeptides, the available literature indicates a great potential of IM for these applications [42].

## Outlook

The study of intact glycopeptides is quickly progressing, due to the advancements in analytical workflows for selective enrichment, improvements in MS instrumentation, and bioinformatics for spectra annotation. Nevertheless, there are open challenges that need to be tackled and that are mostly connected with the complexity of intact glycopeptides. New strategies can be promising to improve the confidence of glycopeptide identification. For instance, retention time prediction exploits the shift of retention times under reversed-phase chromatography, as it depends on the number of monosaccharide units in the glycopeptide. The shifts are predictable and can be exploited to assist the validation of glycopeptide identifications, indicate false identifications, and help in matching of MS/MS spectra with poor fragmentation [46]. New technologies, such as IM, and future developments are still needed for the confident identification of intact glycopeptides. While N-glycosylation is quite common research field in intact glycopeptide analysis, other glycosylation types, such as O-glycosylation (especially on tyrosine), C-mannosylation, and S-glycosylation, are less investigated or poorly studied and need dedicated methods for their analysis.

The complete elucidation of the biological significance of protein glycosylation will need additional techniques. The characterization of intact glycopeptides enables the study of the site occupancy of glycans (macro-heterogeneity) and the variation in glycan species (micro-heterogeneity), but the information on the entire protein is also fundamental to understanding the co-occurrence of glycosylation, by the description of the macro-heterogeneity (site occupancy) and micro-heterogeneity (glycan variations at a specific site) [47]. This has been referred to as meta-heterogeneity [1] and requires the combination of data from bottom-up and high-resolution native MS [48, 49], as recently demonstrated even for proteins with a single glycosylation site, such as ovalbumin, for which more than 150 proteoforms were reported [1, 48]. The combined approach also allows shedding light on the possible co-occurrence with other PTMs and crosstalk. The detailed description of protein glycosylation indicates a large variability, dependent even on a single individual, which represents a new level in personalized proteome profiling and personalized medicine [48]. Finally, it provides a valuable alternative for the quantitative analysis, especially when suppression is a great issue, as the case of sialylated glycopeptides [50], and when glycan distribution and abundance comparison is needed [51].

Most of this trends article described analytical methods developed for, or specifically applied to, mammalian samples. In principle, the described sample preparation strategies and LC–MS/MS analysis can still be used to investigate non-mammalian systems by adapting the data analysis part of the analytical workflow. In fact, databases used for spectra matching usually comprise glycans found in mammals; therefore, they are not suitable for identification of unusual and unexpected glycosylations, such as the ones found in other species. The issue was recently addressed by construction of species-specific glycan structure databases compatible with the commercial software Byonic, showing good results in vegetables and bacteria analysis of both N- and O-glycopeptides [52]. The work demonstrates that the methods described for glycopeptide analysis from mammalian systems can in principle be extended to other types of samples by including the related glycan structures and proteomes. However, it should be noted that the large-scale study by glycoproteomics based on bottom-up analysis of intact glycopeptides of non-mammalian systems and especially of prokaryotes can be hindered because of unknown oligosaccharide chemistries, which imply different oxonium ions in spectra matching and possibly MS/MS acquisition method, and lack of complete protein sequence databases. In addition, although enrichment is applicable, deeper sample fractionation methods, such as gel electrophoresis, may still be necessary [53]. Further efforts will be needed for these applications to match the level of throughput and knowledge available for mammalian samples.
